# Transformed common spatial pattern for motor imagery-based brain-computer interfaces

**DOI:** 10.3389/fnins.2023.1116721

**Published:** 2023-03-07

**Authors:** Zhen Ma, Kun Wang, Minpeng Xu, Weibo Yi, Fangzhou Xu, Dong Ming

**Affiliations:** ^1^School of Precision Instruments and Optoelectronics Engineering, Tianjin University, Tianjin, China; ^2^Academy of Medical Engineering and Translational Medicine, Tianjin University, Tianjin, China; ^3^Beijing Machine and Equipment Institute, Beijing, China; ^4^International School for Optoelectronic Engineering, Qilu University of Technology (Shandong Academy of Sciences), Jinan, China

**Keywords:** brain–computer interface (BCI), electroencephalography (EEG), motor imagery (MI), common spatial pattern (CSP), transformed common spatial pattern (tCSP)

## Abstract

**Objective:**

The motor imagery (MI)-based brain–computer interface (BCI) is one of the most popular BCI paradigms. Common spatial pattern (CSP) is an effective algorithm for decoding MI-related electroencephalogram (EEG) patterns. However, it highly depends on the selection of EEG frequency bands. To address this problem, previous researchers often used a filter bank to decompose EEG signals into multiple frequency bands before applying the traditional CSP.

**Approach:**

This study proposed a novel method, i.e., transformed common spatial pattern (tCSP), to extract the discriminant EEG features from multiple frequency bands after but not before CSP. To verify its effectiveness, we tested tCSP on a dataset collected by our team and a public dataset from BCI competition III. We also performed an online evaluation of the proposed method.

**Main results:**

As a result, for the dataset collected by our team, the classification accuracy of tCSP was significantly higher than CSP by about 8% and filter bank CSP (FBCSP) by about 4.5%. The combination of tCSP and CSP further improved the system performance with an average accuracy of 84.77% and a peak accuracy of 100%. For dataset IVa in BCI competition III, the combination method got an average accuracy of 94.55%, which performed best among all the presented CSP-based methods. In the online evaluation, tCSP and the combination method achieved an average accuracy of 80.00 and 84.00%, respectively.

**Significance:**

The results demonstrate that the frequency band selection after CSP is better than before for MI-based BCIs. This study provides a promising approach for decoding MI EEG patterns, which is significant for the development of BCIs.

## 1. Introduction

Brain–computer interfaces (BCIs) are systems that directly measure brain activities and convert them into artificial outputs. BCIs can replace, restore, enhance, supplement, or improve the natural central nervous system outputs (Birbaumer et al., 2008; Wolpaw and Wolpaw, 2012; Chaudhary et al., 2016; Coogan and He, 2018; Xu et al., 2021; Ju et al., 2022). Currently, scalp electroencephalogram (EEG) is the most popular brain signal for BCIs due to its relatively high temporal resolution and low cost (Park et al., 2012; Xu et al., 2018, 2020; Meng et al., 2020). Among all BCI paradigms, motor imagery (MI)-based BCI is considered more natural than others, which depends on decoding sensorimotor cortex activation patterns induced by imagining movements of specific body parts (Pfurtscheller and Neuper, 2001; Wolpaw et al., 2002).

Event-related desynchronization/synchronization (ERD/ERS) is the most typical EEG feature related to the brain movement intention, which shows a power decrease/increase in specific frequency bands (Pfurtscheller and Da Silva, 1999). For MI-BCIs, it is a key issue to accurately detect the ERD/ERS features. Currently, there are three main categories of MI-BCI algorithms (Herman et al., 2008; Brodu et al., 2011; Lotte et al., 2018), i.e., deep learning-based, Riemannian geometry-based and traditional filtering-based methods. Deep learning (Craik et al., 2019; Zhang et al., 2019) and Riemannian (Fang et al., 2022) methods are recently developed algorithms, both showing good classification performance in MI-BCIs. Deep learning techniques aim to uncover most of the valuable discriminative information within datasets for good classification performance (Goodfellow et al., 2016; Al-Saegh et al., 2021). Both effective features and classifiers are jointly learned directly from the raw EEG. The idea of the Riemannian method is to map the EEG data directly onto a geometrical space equipped with a suitable metric (Barachant et al., 2010, 2012; Yger et al., 2017). Due to its intrinsic nature Riemannian method is robust to noise and provides a good generalization capability (Waytowich et al., 2016; Zanini et al., 2018). The filtering-based methods first filter EEG data in both time and spatial domains and then extract the discriminative features. As a kind of filtering-based method, common spatial pattern (CSP) has been widely used for MI-BCIs due to its conciseness and effectiveness (Ramoser et al., 2000; Lotte et al., 2018). CSP is one of the most efficient and popular methods to extract band-power discriminative features (Ramoser et al., 2000; Blankertz et al., 2007; Chen et al., 2018; Wang et al., 2020). This study aims further to improve the performance of CSP in MI-based BCIs.

The idea of CSP is to maximize the variance of one class and minimize that of the other class simultaneously (Blankertz et al., 2007; Grosse-Wentrup and Buss, 2008; Li et al., 2016; Wang et al., 2020). However, the performance of CSP heavily depends on the selection of EEG frequency bands. It would degrade with inappropriate frequency bands. Previous studies have demonstrated a great deal of ERD/ERS variability among subjects regarding their frequency characteristics (Pfurtscheller et al., 1997; Guger et al., 2000). To address this problem, researchers have proposed several advanced versions of CSP to optimize the selection of frequency bands before applying CSP. For example, Lemm et al. (2005) designed the common spatio-spectral pattern (CSSP) algorithm, which could individually tune frequency filters at each electrode position. However, the frequency filter setting in CSSP is inflexible (Dornhege et al., 2006). Dornhege et al. (2006) proposed the common sparse spectral spatial pattern (CSSSP), which could simultaneously optimize a finite impulse response (FIR) filter and a spatial filter to select the individual-specific frequency bands automatically. It yielded better performance than CSSP, but the optimization process of CSSSP is complicated and time-consuming. Later, Novi et al. (2007) tried to decompose the EEG signals into sub-bands using a filter bank instead of temporal FIR filtering, called sub-band common spatial pattern (SBCSP). SBCSP could mitigate the time-consuming problem of the fine-tuning process during the construction of BCI classification models. In 2008, the filter bank common spatial pattern (FBCSP) was proposed by Kai Keng et al. (2008), which had the best performance in frequency band selection (Ang et al., 2012).

Besides, the regularization method has also been studied to further boost the performance of CSP. Lu et al. (2009) proposed the regularized common spatial pattern (R-CSP), which regularized the covariance matrix estimation for typical CSP. Park and Lee (2017) utilized principal component analysis (PCA) to extract R-CSP features from all frequency sub-bands, called sub-band regularized common spatial pattern (SBRCSP). Later, Park et al. (2017) proposed the regularized filter bank common spatial pattern (FBRCSP), which combined R-CSP with the filter bank structure. Both SBRCSP and FBRCSP showed better performance than CSP, FBCSP, and R-CSP.

This study proposed a novel algorithm called transformed common spatial pattern (tCSP) to further improve the selection of optimal frequency bands. Unlike traditional approaches that optimize the frequency selection before CSP filtering, the proposed tCSP selects the subject-specific frequency after CSP filtering. Two offline datasets and an online evaluation were employed to verify the effectiveness of tCSP.

## 2. Materials and methods

### 2.1. Dataset description

This study uses two offline datasets to evaluate the performance of the proposed algorithms. One is the dataset (not publicly available) from an experiment performed by our team. This experiment is called experiment one in this paper. The other is the Dataset IVa of BCI Competition III (Blankertz et al., 2006), which is always used for testing CSP-based algorithms. Moreover, we performed the second experiment, i.e., experiment two, to assess the effectiveness and suitability of the proposed algorithm in the online operation.

#### 2.1.1. The dataset of experiment one

**FIGURE 1:**
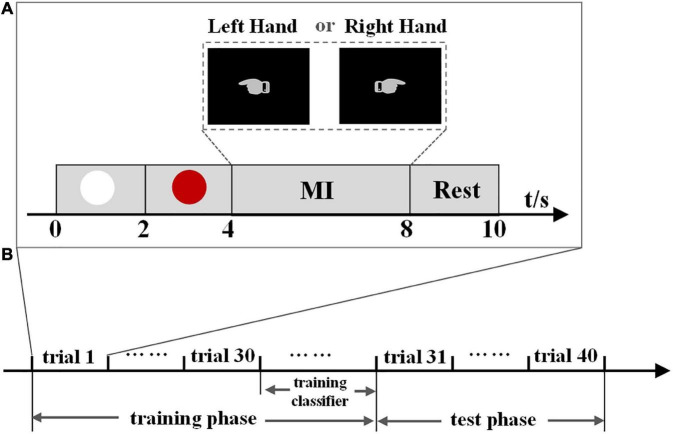
**(A)** Timing of a trial paradigm of experiment one and experiment two. A white circle is displayed for 2 s at the beginning, then a red circle appears 2 s later, reminding the subject to be prepared for an experiment task. From the 4 to 8 s, the subject performs the MI task. A hand pointing to the left indicates the left-hand MI task and pointing to the right indicates the right-hand MI task. In the test phase of experiment two, subjects were informed of the output after each MI task. Finally, the subject is asked to keep a resting state for 2 s. **(B)** Experiment phases of experiment two. We use the proposed methods to train a classifier in the training phase, then evaluate its performance in the test phase.

The dataset contains EEG signals from eleven healthy subjects aged 21–26 years. In the experiment, they were required to perform two different MI tasks of left- and right-hand movements. Figure 1A shows the timing of a trial paradigm. An electrode placed on the nose served as the reference, and the ground electrode was placed on the forehead. The data were acquired by a SynAmps2 system with a 64-channel EEG quick-cap, and 60 channels were measured at positions of the international 10/20-system. The data were sampled at 1000 Hz, band-pass filtered between 0.5 and 100 Hz. A notch filter with 50 Hz was also used to remove the power grid noise during the data acquisition. The experiment was composed of 10 blocks, and each block consisted of 8 trials (4 trials for each hand). The sequence of cues for different MI tasks was presented randomly in each block. The experiment was approved by the ethical committee of Tianjin University.

#### 2.1.2. Dataset IVa of BCI competition III

The Dataset IVa of BCI Competition III was recorded from five healthy subjects. This dataset contains two MI tasks, i.e., the right-hand and right-foot movements. The data were captured by a BrainAmp amplifier system with a 128-channel Ag/AgCl electrode cap, and 118 EEG channels were measured at positions of the extended international 10/20-system. The data were sampled at 1,000 Hz and then band-pass filtered between 0.05 and 200 Hz. There were 280 trials for each subject, namely 140 trials for each task. Table 1 shows the number of trials for training and test data for the five subjects. In each trial, a visual cue was shown for 3.5 s, and the subjects performed the MI task. The presentation of target clues was intermitted by periods of random length from 1.75 to 2.25 s so that the subjects could take a short break. In this dataset, the training and test sets consisted of different sizes for each subject.

**TABLE 1:** The number of training and test trials for each subject of the dataset IVa.

Subject	Training trials	Test trials
aa	168	112
al	224	56
av	84	196
aw	56	224
ay	28	252

#### 2.1.3. Online evaluation

The most effective BCI research usually incorporates offline and online evaluations (Wolpaw and Wolpaw, 2012). We performed the experiment two to evaluate the performance of the proposed algorithm in the online operation. Ten right-handed and healthy subjects (four males and six females, aged 22–32) participated in the experiment. Four subjects had no prior experience with MI-based BCIs. All the subjects signed a consent form in advance. The purpose and procedure of the experiment were clearly explained to each subject before the EEG recording. The study was approved by the ethical committee of Tianjin University.

Figure 1A shows the timing of a trial paradigm, which is the same as experiment one. A hand pointing to the left indicates the left-hand MI task, and pointing to the right indicates the right-hand MI task. During the experiment, subjects were seated in a chair about 1 m from a monitor that displayed the task cues on a black background. The subjects were required to perform left- and right-hand MI tasks. Experiment two consisted of training and test phases (Figure 1B), containing forty trials. The training phase contained 30 trials (15 for each hand) used as the training dataset to generate individual classification parameters. The data in the test phase, containing five trials for each hand, was used as the test dataset to evaluate the performance of the proposed method. The BCI system operated in real-time in experiment two. In the test phase, subjects were informed of the output after each MI task so that they could adjust their brain signals to ensure that the correct intent could be continuously accomplished in the following MI tasks. The sequence of cues for different tasks was presented randomly in the training and test phases. An electrode placed on the vertex served as the reference, and the ground electrode was placed on the forehead. The EEG data were acquired by a Neuroscan SynAmps2 system with a 64-channel quick-cap using the international 10–20 system, and data were sampled at 1,000 Hz.

### 2.2. Preprocessing

First, the EEG data were subjected to a band-pass FIR filter to remove slow signal drifts and high-frequency noise and down-sampled to 100 Hz. Then, the data were processed by the common average reference (CAR) and extracted to form an epoch. Finally, we divided each dataset into training, calibration and test data.

For the datasets from experiment one and experiment two, the EEG data were band-pass filtered from 8 to 32 Hz. The data between 0.5 and 3.0 s, with respect to cue onset, were extracted for classification. For Dataset IVa, we filtered the data from 7 to 30 Hz and extracted the data between 0.5 and 2.5 s with respect to cue onset for classification. For evaluating the performance of the proposed algorithm, we used a 10-fold cross-validation method for the dataset of experiment one and a 5-fold cross-validation method for Dataset IVa. For experiment one, eight-tenths of the data were used as training data and the remaining two-tenths were divided equally as calibration and test data. For Dataset IVa, three-fifths of the data were used as training data, one-fifths as calibration data and the remaining one-fifths as test data. We used a 10-fold cross-validation method for the training classifier in the training phase of experiment two. Nine-tenths of the data from the training phase were used as training data and the remaining data were used as calibration data. The data from the test phase were used as test data.

### 2.3. CSP filtering

We performed spatial filtering using the CSP on the preprocessed data. The CSP filter *W* can be obtained using the training data of two classes. The EEG data can be presented as a matrix $E_{n}^{i}\in R^{N\times T}$, where *i* denotes the *i*-th trial, *n* (i.e., 1,2) denotes each of the two MI tasks, *N* is the number of channels, and *T* is the number of samples per channel. The CSP method can be summarized using the following steps.

Firstly, the data were decentered as follows:


(1) $$E_{n}^{i}=E_{n}^{i}-\bar{E_{n}}$$

where *n* = 1,2 and $\bar{{\text{E}}_{\text{n}}}$ is the average over the trials of each group.

Secondly, we calculated the normalized spatial covariance of $E_{n}^{i}$, which can be obtained from:


(2) $$\bar{C_{n}}=\frac{1}{I}\,\sum_{i}^{I}\frac{E_{n}^{i}\cdot \left(E_{n}^{i}\right)\prime }{t\,r\,a\,c\,e\,\left(E_{n}^{i}\cdot \left(E_{n}^{i}\right)\prime \right)}$$

where (⋅)’ denotes the transpose operator, trace(*X*) is the sum of the diagonal elements of *X* and $\bar{{\text{C}}_{\text{n}}}\in R^{N=T}$ denotes the average spatial covariance of all trials for class *n*. The composite spatial covariance is given as:


(3) $$C_{c}=\bar{C_{1}}+\bar{C_{2}}$$

where the subscript *c* is short for composite. Thirdly, we calculated the whitening transformation matrix. The process of eigenvalue decomposition on *C*_*c*_ is shown below:


(4) $$C_{c}=V_{c}\,D_{c}\,V_{c}^{\prime }$$

where *V*_*c*_ is the matrix of eigenvectors and *D*_*c*_ is the diagonal matrix of eigenvalues sorted in descending order, and the whitening transformation matrix is presented as:


(5) $$P=\sqrt[{-\frac{1}{2}}]{D_{c}}\,V_{c}^{\prime }$$

Fourthly, we performed a whitening transformation:


(6) $$S_{n}=P\,\bar{C_{n}}\,P^{\prime }$$

then *S*_1_ and *S*_2_ share the same eigenvectors, and they can be factored as:


(7) $$S_{n}=B\,\Lambda_{n}\,B^{\prime }$$

where *B* is the matrix of eigenvectors and *Λ_*n*_* (*n* = 1,2) is the diagonal matrix of eigenvalues, which are sorted in descending order. The eigenvectors with the largest eigenvalues for *S*_1_ had the smallest eigenvalues for *S*_2_ and vice versa. The spatial filter can be expressed as:


(8) $$W=B^{\prime }\,P$$

Finally, with the spatial filter *W*, the original EEG can be transformed into uncorrelated components:


(9) $$Z^{i}=W\prime \,E^{i}$$

where *i* denotes the *i*-th trial. For each of the two imagery movements, the variances of only a few signals that correspond to the first and last *M* eigenvalues are most suitable for discrimination. Hence, after spatial filtering, we got the data *Z^i^* ∈ *R*^2*M*=*T*^. We selected the first and last four eigenvectors of the *W* for feature extraction, i.e., the *M* was set to 4 in this paper. The CSP features are calculated as:


(10) $$\sigma^{p}=l\,o\,g\,\left(\frac{v\,a\,r\,\left(z^{p}\right)}{\sum_{p=1}^{2\,M}v\,a\,r\,\left(z^{p}\right)}\right),p=1,2,\cdots ,2\,M$$

where *Z* denotes the transformed data in Equation 9, and the log transformation, i.e., the *log*(⋅) in Equation 10, approximates the normal distribution of the data. We finally get 2*M* features for one trial, forming a feature vector *y*_*c*_.

### 2.4. Data transformation and concatenation

After CSP spatial filtering (Equation 9), we transferred the data *Z^i^* to the time-frequency domain with Morlet wavelet to present more discriminative information. The time-frequency transferred data can be presented as a matrix *G^i^∈R^2M × J × T^*, where *J* denotes the number of frequency points. Then we concatenated the transferred data in the time dimension, i.e., the data *G^i^* was reconstituted to a matrix *H^i^* ∈ *R*^*J*×*T*_*w*_^, where *T_*w*_* = *T ×* 2*M*.

### 2.5. Feature extraction and pattern classification

In this section, we first extracted tCSP features from the concatenated data *H^i^* at all frequency points. Then we selected the optimal frequency point for classification by the calibration procedure, which was a process of data-dimension reduction to remove redundant frequency information for each subject. Furthermore, CSP features were extracted and combined with tCSP features to further enhance the classification performance of BCI systems.

#### 2.5.1. tCSP feature extraction

Transformed common spatial pattern feature consists of Pearson correlation coefficients ρ. To extract tCSP features, we first selected the data at frequency point *j* from the matrix *H*^*i*^ of training data, which can be presented as a vector *K*^*i*,*j*^ ∈ *R*^*T_w_*^. Then we calculated the templates according to:


(11) $$t\,e\,m\,p\,l\,a\,t\,e_{n}^{j}=\frac{1}{I}\,\sum_{i=1}^{I}K_{n,t\,r}^{i,j},n=1,2$$

where *I* denotes the number of training-data trials, *i* denotes the *i*-th trial, *n* denotes each of the two MI tasks, *tr* denotes the training data. Finally, we calculated tCSP features of all data according to:


(12) $$\rho^{i,j}=\left[\begin{array}{c} c\,o\,r\,r\,\left(t\,e\,m\,p\,l\,a\,t\,e_{1}^{j},K^{i,j}\right)\\ c\,o\,r\,r\,\left(t\,e\,m\,p\,l\,a\,t\,e_{2}^{j},K^{i,j}\right) \end{array}\right]$$

where *corr*(⋅) is Pearson-correlation calculating, *ρ*^i,j^** denotes the tCSP features of the *i*-th trial at the frequency point *j*.

#### 2.5.2. Fisher discriminant analysis

In this paper, fisher discriminant analysis (FDA) (Mika et al., 1999) was used for pattern classification. FDA is a classical classifier that maximizes the ratio between inter-class and intra-class variance. FDA classifier is mainly based on the decision function defined as follows:


(13) $$f\,\left(y\right)=U^{\prime }\,y+\omega_{0}$$

where *y* is the feature vector obtained from the above steps, *U* is a weight vector, and ω_0_ is a threshold. The values of the weight vector and the threshold are identified by employing fisher’s criterion on the training data. The classification process is based on the separation by the hyperplane as described in the following:


(14) $$\left\{ \begin{array}{c} f\,\left(y\right)>0,y\,\epsilon \,D_{1}\\ f\,\left(y\right)<0,y\,\epsilon \,D_{2} \end{array}\right.$$

where *D*_1_ and *D*_2_ are two different classes. In this study, the method of *N*-fold cross-validation was applied to evaluate the classification performance of the proposed method.

#### 2.5.3. tCSP feature selection

We utilized the calibration data to find out the subject-specific optimal frequency points for classification. Concretely, the calibration features were used as the inputs of an FDA classifier, which was trained by training data, and then we got a classification accuracy matrix *Q*∈*R*^*A ×J*^, where *A* indicates the number of subjects, and *J* indicates the number of the frequency points. To get stable and reliable results, we applied 10-fold cross-validation to calculate the classification accuracy at each frequency point. We used a sparse matrix *F*∈*R*^*A* ×^*^P^* to select the optimal frequency point *F*_*a*_ as:


(15) $$F_{a}=Q\cdot F^{\prime }$$

In the sparse matrix *F*, the elements where the highest classification accuracies occur in the calibration process were set to one for each subject, and the others were set to zero. Then the training and test features at *F*_*a*_ were extracted. Finally, we got a tCSP feature vector *y*_*t*_ for each subject.

#### 2.5.4. Feature combination

To further improve the performance of the MI-based BCI, we combined the selected tCSP features and CSP features, getting a fusion feature vector *Y*=[*y*_*t*_,*y*_*c*_]′, to provide more discriminative information for classification. The fusion features were used to evaluate the performance of the proposed method.

**FIGURE 2:**
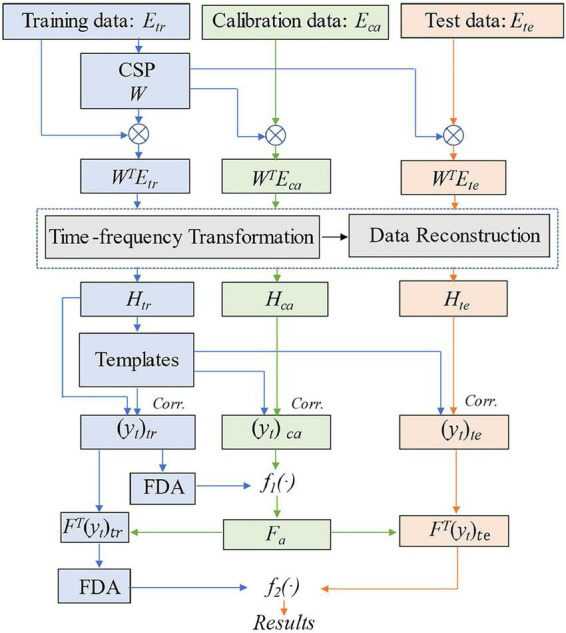
Flow chart of the proposed tCSP. *tr* indicates training data, *ca* indicates calibration data, *te* indicates test data, *H* indicates the concatenated time-frequency data, *Corr.* indicates Pearson correlation, *y*_*t*_ indicates the tCSP feature, *F*_*a*_ indicates the optimal frequency point, *f_1_(⋅)* and *f_2_(⋅)* indicate decision functions.

The main processes of the method are illustrated in Figure 2, and the pseudocode of tCSP is shown in the Appendix.

## 3. Results

### 3.1. ERD patterns of left- and right-hand MI tasks

The mu (8–14 Hz) and beta (14–30 Hz) ERDs reflect the brain oscillation patterns induced by MI. Event-related spectral perturbation (ERSP) measures the mean dynamic changes from baseline in terms of the power spectrum over time in a broad frequency range (Makeig, 1993; Delorme and Makeig, 2004; Makeig et al., 2004). It can provide detailed information on ERD/ERS patterns. Hence, we first analyzed the ERD patterns between the frequency range of 8–32 Hz and the time range of −1 to 5 s for each MI task by ERSP. The baseline is the mean of the data ranging from −1s to 0 s. The average ERSP values of electrodes C3 and C4 are compared for the left- and right-hand MI tasks.

**FIGURE 3:**
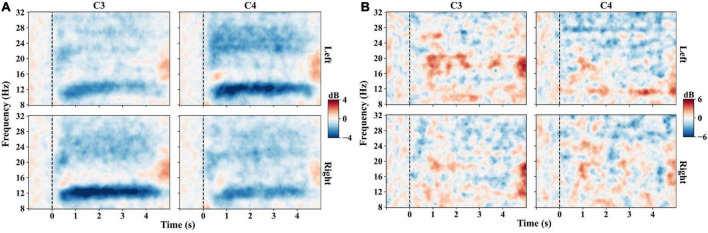
Time-frequency maps on C3 and C4 channels of experiment one. Left indicates the left-hand MI task, and right indicates the right-hand MI task. Blue indicates the ERD, red indicates the ERS, and black dashed lines indicate the task onset. **(A)** Time-frequency maps averaged across trials of all subjects. The left-hand MI task induces a stronger mu-band ERD on C4 than on C3, and the right-hand MI task induces a stronger ERD on C3. **(B)** Time-frequency maps averaged across trials of subject S4, showing little ERD patterns when performing MI tasks.

Figure 3A presents the averaged time-frequency maps of C3 and C4 across all the subjects of experiment one. The EEG power decreased after the zero-time point when the subjects performed the MI tasks, especially in the frequency range of 8–14 Hz, which refers to ERD. In addition, the phenomenon of contralateral dominance is distinctly observed in Figure 3A. The ERD of the mu band (8–14 Hz) is more significant at C4 than C3 for the left-hand MI task. On the contrary, the right-hand MI task induces lower ERD at C3. However, not all the subjects show distinguishable ERD patterns, such as the time-frequency patterns of subject 4 in Figure 3B.

### 3.2. tCSP feature extraction

**FIGURE 4:**
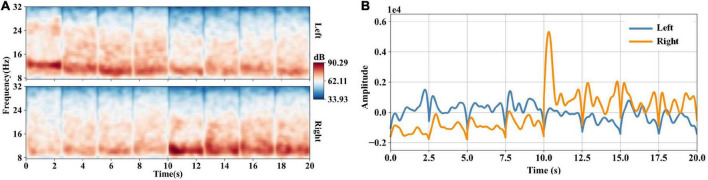
**(A)** Concatenated time-frequency maps averaged across trials of subject S9. After CSP filtering, we obtain eight channels’ data with a time window of 2.5 s. We transform the CSP-filtered data into the time-frequency domain and get eight time-frequency data segments. Then we concatenate the eight time-frequency data segments, forming the concatenated time-frequency data of 20 s. **(B)** Waveforms of the templates at the optimal frequency point (11.87 Hz) of subject S9. After data concatenation, the optimal frequency points for classification are selected through the method of section “2.5.3. tCSP feature selection”. The templates are calculated using training concatenated time-frequency data at the optimal frequency point.

After CSP filtering and time-frequency transformation, we obtained eight time-frequency data segments with a time window of 2.5 s. Figure 4A shows the concatenated time-frequency maps averaged across trials of subject S9. The four segments before 10 s correspond to the first four eigenvectors of CSP filter *W*, and the last four segments correspond to the last four eigenvectors of *W*. The frequency ranges from 8 to 32 Hz. We can observe the spectral power increase in the mu band induced by the left- and right-hand MI tasks. Figure 4B shows waveforms of the templates at the optimal frequency point of subject S9. The templates are calculated according to Equation 10. The templates show a distinct discriminative ability for the left- and right-hand MI tasks.

**FIGURE 5:**
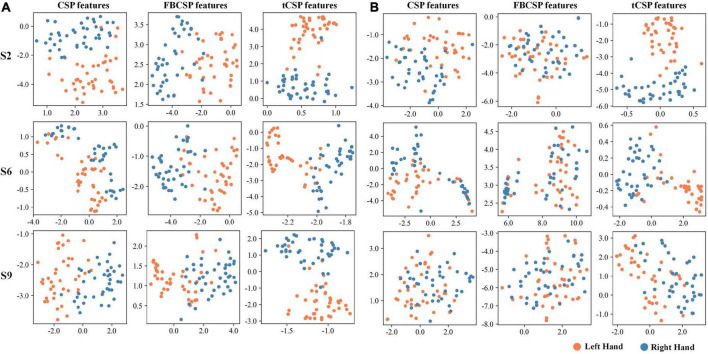
Feature visualization of different methods using t-SNE. Orange dots indicate samples of left-hand MI tasks, blue dots indicate right-hand MI tasks. The training processes used 40 samples **(A)** and eight **(B)**.

We also visualized the features to understand the proposed method’s effect further. Figure 5 displays the feature distribution maps transformed by t-SNE (Van der Maaten and Hinton, 2008) for subjects S2, S6, and S9 from experiment one. The features were extracted by CSP, FBCSP, and tCSP with 40 training samples (Figure 5A) and 8 training samples (Figure 5B) for each subject. The tCSP features of different MI tasks are more diverse than those of CSP and FBCSP. The two tasks could be better separated with more training samples for all methods.

### 3.3. Optimal frequency points selection

**FIGURE 6:**
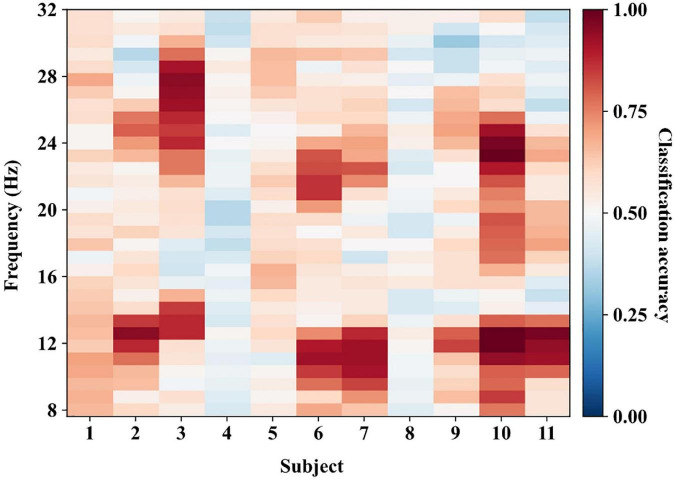
The classification performance in the calibration process of experiment one. We calculated the classification accuracies using calibration data at all frequency points. The frequency points with the highest classification accuracy are selected as the optimal frequency points for each subject.

We transferred the data ranging from 8 to 32 Hz to the time-frequency domain with a step of about 0.8 Hz, generating 32 frequency points for each subject. In the calibration process, we first calculated the classification accuracies of all frequency points for each subject using the calibration data with a 10-fold cross-validation approach. Figure 6 shows the classification performance for all subjects of experiment one. The darker red color denotes higher classification accuracy. Then we selected the frequency points with the highest classification accuracy as the optimal frequency points *F*_*a*_ for each subject. Finally, we tested the performance of the proposed methods using the test data at *F*_*a*_. Figure 6 shows that most optimal frequency points are distributed in the mu and beta bands with individual variation.

### 3.4. Classification performance of experiment one

Table 2 summarizes the classification accuracies of CSP, FBCSP, tCSP, and the combination methods. All the methods shared the same parameters, such as the frequency band and time window for feature extraction. To get reliable experimental results, we used a 10-fold cross-validation approach. The highest classification accuracy was highlighted in bold for each row in the table. One-way repeated measures ANOVAs were employed to indicate whether the accuracy differences among methods reached the statistical significance level.

**TABLE 2:** The classification accuracies (%) of all subjects from experiment one using CSP, FBCSP, tCSP and the combination method.

Subjects	CSP	FBCSP	tCSP	CSP+FBCSP	tCSP+FBCSP	tCSP+CSP
S1	68.75	65.00	65.00	56.25	65.00	**71.25**
S2	82.50	88.75	**96.25**	90.00	**96.25**	**96.25**
S3	83.75	97.50	**98.75**	**98.75**	97.50	98.25
S4	42.50	**56.25**	53.75	**56.25**	54.50	55.25
S5	62.50	65.00	61.25	63.75	62.50	**66.25**
S6	76.25	88.75	96.25	92.50	**97.50**	**97.50**
S7	91.25	91.25	93.75	90.00	90.00	**95.00**
S8	56.25	61.25	**66.25**	58.75	68.75	65.25
S9	71.25	72.50	**90.00**	72.50	81.25	**90.00**
S10	97.50	98.75	**100**	**100**	**100**	**100**
S11	97.50	86.25	98.75	98.75	**100**	97.50
Av.	75.45	79.20	83.64	79.77	83.02	**84.77**
Std.	17.39	15.46	17.97	18.33	17.28	16.67

The best result of each subject is set in bold.

Mauchly’s test indicated that the assumption of sphericity was violated. Hence, Correction was done using the Greenhouse–Geisser criterion. The results revealed that the accuracy differences were significant for all the methods [*F*(2.84, 28.37) = 7.06, *p* < 0.01]). The tCSP method got an average accuracy of 83.64%, which was significantly better than that obtained by CSP (*t*_10_ = 3.28, *p* < 0.01) and FBCSP (*t*_10_ = 2.29, *p* < 0.05). The combination method of tCSP and CSP achieved an average accuracy of 84.77%, yielding statistically better performance than CSP (*t*_10_ = 4.24, *p* < 0.01), FBCSP (*t*_10_ = 3.38, *p* < 0.01), and CSP+FBCSP (*t*_10_ = 2.63, *p* < 0.05). The performance of tCSP+FBCSP was significantly better than CSP+FBCSP (*t*_10_ = 2.36, *p* < 0.05). At the same time, there was no significant difference between tCSP and the combination methods of tCSP+CSP and tCSP+FBCSP (*p* > 0.05).

**FIGURE 7:**
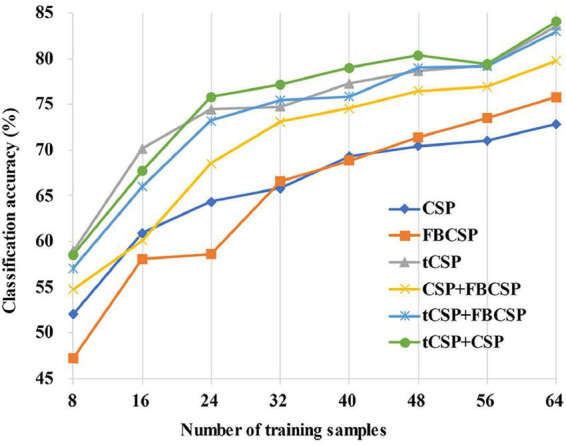
The variation trend of average classification accuracies with different training sample sizes. The classification accuracies rise with the increase of training samples for all methods. The proposed methods achieve better classification performance than the others, even in small-sample setting conditions.

Figure 7 shows the average classification accuracies across all subjects with the different number of training samples for all methods. The classification accuracies had a rising trend with the increase of training samples. It should be noted that tCSP achieved significantly better performance than CSP and FBCSP for all conditions. The tCSP+CSP method achieved the best performance when the number of training samples was between 24 and 56.

### 3.5. Classification performance of dataset IVa

Table 3 compares the classification results of the proposed methods with some other CSP-based approaches. In this study, the raw data were bandpass filtered between 7 and 30 Hz. The data from 0.5 to 2.5 s after cue onset were selected for feature extraction and classification. A 5-fold cross-validation was applied to evaluate the classification performance of the proposed method. tCSP achieved an average classification accuracy of 91.33%, and the combination method of tCSP and CSP achieved an average classification accuracy of 94.31%, with two subjects achieving 100% accuracy, which obtained the highest average classification accuracy.

**TABLE 3:** Comparison of the classification performance for dataset IVa in BCI competition III.

References	Feature extraction	Classification method	Accuracy (%)					
			**aa**	**al**	**av**	**aw**	**ay**	**Av.**
Song and Epps, 2007	CSP, dynamic features	SVM	91.50	99.20	70.90	**96.90**	94.70	90.60
Song and Epps, 2007	CSSP	SVM	85.40	97.70	67.70	96.50	94.00	88.26
Song and Epps, 2007	CSSSP	SVM	88.40	97.90	68.20	93.50	89.50	87.50
Novi et al., 2007	SBCSP, Bayesian	SVM	89.30	98.60	70.40	95.70	95.70	90.00
Yong et al., 2008	SCSP	LDA	57.50	86.90	54.40	84.40	84.30	73.50
Kai Keng et al., 2008	FBCSP	MIBIF	−	−	−	−	−	90.30
Meng et al., 2009	CSP, channel selection	SVM	82.40	98.60	76.80	94.00	96.60	89.68
Lu et al., 2010	R-CSP	Aggregation	76.80	98.20	74.50	92.90	77.00	83.90
Park et al., 2017	FBRCSP	ensemble method	91.07	94.64	75.00	76.78	93.65	86.23
Park and Lee, 2017	SBRCSP	FLD	86.81	98.21	63.78	89.05	77.78	82.69
Park and Chung, 2019	LRFCSP	SVM	**98.93**	93.21	81.79	93.21	97.50	92.93
This work	tCSP	FDA	87.06	97.39	75.56	96.67	**100**	91.33
This work	tCSP, FBCSP	FDA	85.88	97.39	77.78	96.67	**100**	91.54
This work	tCSP, CSP	FDA	88.24	**100**	**86.67**	96.67	**100**	**94.31**

The best result of each subject is set in bold.

### 3.6. Results of the online evaluation

**FIGURE 8:**
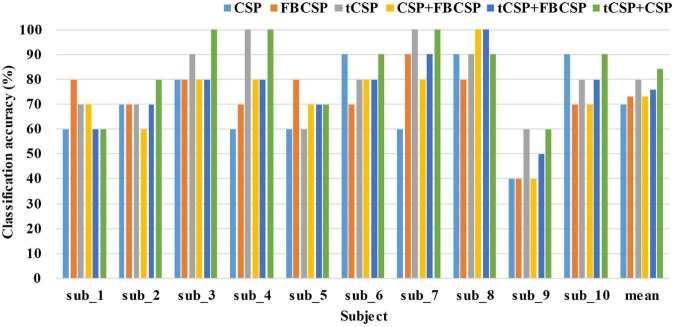
The classification accuracies of different methods in experiment two. The mean indicates the average classification accuracies across all the subjects. tCSP or tCSP+CSP achieves the highest classification accuracies for most subjects.

The classification parameters in experiment two, such as frequency ranges and the sampling rate, were selected in accordance with experiment one. Furthermore, we also performed the pseudo-online evaluation of CSP, FBCSP, CSP+FBCSP and tCSP+FBCSP using the same data collected from experiment two. Figure 8 shows the classification results of all subjects. The accuracy differences were significant for all the methods [*F*(5, 45) = 3.27, *p* < 0.05]. tCSP achieved an average accuracy of 80.00%, with two subjects getting an accuracy of 100%. The combination method of tCSP and CSP achieved an average accuracy of 84.00%, with three subjects getting an accuracy of 100%, which was significantly better than that obtained by CSP (*t*_9_ = 2.81, *p* < 0.05), FBCSP (*t*_9_ = 2.28, *p* < 0.05), CSP+FBCSP (*t*_9_ = 2.70, *p* < 0.05), and tCSP+FBCSP (*t*_9_ = 2.75, *p* < 0.05). There was no significant difference between the classification accuracies of tCSP and CSP or FBCSP (*p* > 0.05).

## 4. Discussion

As a typical algorithm for ERD-feature extraction, CSP heavily depends on selecting frequency bands. However, not all subjects show distinct ERD patterns with strong discriminant ability when performing MI tasks (Figure 3B). CSP features generally yield poor classification performance with an inappropriate frequency band (Novi et al., 2007). Hence, selecting appropriate subject-specific frequency ranges before CSP is an effective and popular measure to improve the performance of MI-based BCIs (Novi et al., 2007; Kai Keng et al., 2008; Park and Lee, 2017; Park et al., 2017). This study proposed tCSP method to optimize the frequency selection after CSP filtering, achieving significant better performance than the traditional CSP methods.

tCSP addresses the MI-induced EEG features in both spatial and frequency domains. After spatial filtering by CSP, we increased the dimension of the data, i.e., the time-frequency transformation and data concatenation, aiming to present more detailed discriminative information in the time-frequency domain (Figure 4A). Then, we reduced the dimension of the data by selecting the optimal subject-specific frequency points for classification. The data dimension increasing and reducing processes may reinforce features’ discriminability. As a result, from Figure 5, we can see that the distribution of tCSP features had a more obvious divergence with better discriminability than CSP and FBCSP, especially when the number of training samples was limited.

CSP features reflect a broad frequency-band power variation of MI EEG data (Ramoser et al., 2000; Kai Keng et al., 2008). In contrast, tCSP extracts feature from frequency point ranges, which may get finer discriminative information in the frequency domain than CSP. Thus, tCSP got better performance than CSP. The tCSP and CSP features may reflect different fineness levels of frequency optimization, so the combination of tCSP and CSP may provide comprehensive discriminative information to further improve the performance of MI classification. From the results in Table 2, the tCSP+CSP method got the best performance on average with the highest accuracy of 100%, which was significantly better than CSP, FBCSP, and CSP+FBCSP. For dataset IVa (Table 3) and the online evaluation (Figure 8), the combination of tCSP and CSP got the best performance on average.

Generally speaking, a limited number of training samples would bring about a high variance for the covariance estimation, which might result in a biased estimation of eigenvalues (Friedman, 1989). Thus, a small-sample setting condition usually results in poor performance of classifiers. From the results of this study, the proposed method performed relatively well in small-sample setting conditions. For the dataset of experiment one (Figure 7), the tCSP method achieved an average accuracy of about 70% with 16 training samples, approximately equal to that of traditional CSP and FBCSP with 48 training samples.

## 5. Conclusion

This study designed a novel feature extraction method, i.e., tCSP, to optimize the frequency selection after CSP filtering. tCSP could achieve better performance than the traditional CSP and filter bank CSP. Furthermore, the combination of tCSP and CSP could extract more discriminative information and further improve the performance of MI-based BCIs. The results of a dataset collected by our team, a public dataset and an online evaluation verified the feasibility and effectiveness of the proposed tCSP. In general, optimizing the frequency selection after CSP is a promising approach to enhance the decoding of MI EEG patterns, which is significant for the development of BCIs.

## Data availability statement

The raw data supporting the conclusions of this article will be made available by the authors, without undue reservation.

## Ethics statement

The studies involving human participants were reviewed and approved by the Ethical Committee of Tianjin University. The patients/participants provided their written informed consent to participate in this study.

## Author contributions

ZM, KW, MX, and DM conceived the study. ZM and WY designed and conducted the experiments. ZM performed data analyses. ZM and KW wrote and edited the initial draft. MX, FX, and DM performed proofreading and finalizing of the manuscript. All authors contributed to the article and approved the submitted version.

## Appendix

Here is the pseudocode of the tCSP:

Algorithm: tCSP

Input: EEG dataset *E*∈*R*^*N*×*T*^.

Output: The tCSP feature vector *y*_*t*_.

Step 1 (Preprocessing):

• Divide the dataset into training, calibration and test data.

Step 2 (CSP Filtering):

• Center the training data as $E_{n}^{i}=E_{n}^{i}-\bar{E_{n}}$, where *n =* 1,2 and *i* denotes the *i*-th trial.
• Calculate composite spatial covariance $C_{c}=\bar{C_{1}}+\bar{C_{2}}$.
• Eigenvalue decomposition: $C_{c}=V_{c}\,D_{c}\,V_{c}^{\prime }$.
• Calculate $P=\sqrt[{-\frac{1}{2}}]{D_{c}}\,V_{c}^{\prime }$.
• Whitening transformation: $S_{n}=P\,\bar{C_{n}}\,P^{\prime }$.
• Eigenvalue decomposition: Sn=B⁢Λ⁢B′n.
• Calculate *W*=*B*′*P*.
• Transform all the data as *Z^i^*=*W*′*E^i^*.

Step 3 (Data transformation and concatenation):

• Transfer the data *Z^i^* to the time-frequency domain *G^i^*∈*R*^*2M* × *J* × *T*^.
• Reconstitute the data *G^i^* to a matrix *H^i^* ∈ *R*^*J*×*T*_*w*_^, where *T_*w*_* = *T ×* 2*M*.

Step 4 (feature extraction):

• For *j* = 1: *J*
  1. Select the data *K*^*i*,*j*^ ∈ *R^T^*_*w*_ from *H^i^* at frequency point *j*
  2. Calculate $t\,e\,m\,p\,l\,a\,t\,e_{n}^{j}=\frac{1}{I}\,\sum_{i=1}^{I}K_{n}^{i,j}$ using the training data
  3. Calculate tCSP features as $\rho^{i,j}=\left[\begin{array}{c} c\,o\,r\,r\,\left(t\,e\,m\,p\,l\,a\,t\,e_{1}^{j},K^{i,j}\right)\\ c\,o\,r\,r\,\left(t\,e\,m\,p\,l\,a\,t\,e_{2}^{j},K^{i,j}\right) \end{array}\right]$
  4. Train an FDA classifier using training features, then calculate a classification accuracy using calibration features
  5. All the classification accuracies form a matrix *Q*∈*R*^*A*^^×^^*J*^, where *A* indicates the number of subjects
  6. Break and go to next step
• Select the frequency points with the highest classification accuracies as *F*_*a*_=*Q*⋅*F*′, where *F* is a sparse matrix.
• Select the tCSP features at *F_a* to form a feature vector *y*_*t*_.
